# In Vitro Characterization of In Situ Alloyed Ti6Al4V(ELI)-3 at.% Cu Obtained by Laser Powder Bed Fusion

**DOI:** 10.3390/ma14237260

**Published:** 2021-11-27

**Authors:** Anna Martín Vilardell, Pavel Krakhmalev, Ina Yadroitsava, Igor Yadroitsev, Natalia Garcia-Giralt

**Affiliations:** 1 Department of Engineering and Physics, Karlstad University, 651 88 Karlstad, Sweden; pavel.krakhmalev@kau.se; 2 Department of Mechanical Engineering and Mechatronics, Central University of Technology, Bloemfontein 9300, Free State, South Africa; iyadroitsava@cut.ac.za (I.Y.); iyadroitsau@cut.ac.za (I.Y.); 3 IMIM (Institut Hospital del Mar d’Investigacions Mèdiques), CIBERFES, ISCIII, Doctor Aiguader 88, 08003 Barcelona, Spain; ngarcia@imim.es

**Keywords:** laser powder bed fusion, Ti–Cu alloys, in-vitro tests, surface roughness, implants

## Abstract

The intensive cytotoxicity of pure copper is effectively kills bacteria, but it can compromise cellular behavior, so a rational balance must be found for Cu-loaded implants. In the present study, the individual and combined effect of surface composition and roughness on osteoblast cell behavior of in situ alloyed Ti6Al4V(ELI)-3 at.% Cu obtained by laser powder bed fusion was studied. Surface composition was studied using scanning electron microscopy, energy dispersive spectroscopy, and X-ray diffraction. Surface roughness measurements were carried out using confocal microscopy. In vitro osteoblast performance was evaluated by means of cell morphology observation of cell viability, proliferation, and mineralization. In vitro studies were performed at 1, 7, and 14 days of cell culture, except for cell mineralization at 28 days, on grounded and as-built (rough) samples with and without 3 at.% Cu. The addition of 3 at.% Cu did not show cell cytotoxicity but inhibited cell proliferation. Cell mineralization tends to be higher for samples with 3 at.% Cu content. Surface roughness inhibited cell proliferation too, but showed enhanced cell mineralization capacity and therefore, higher osteoblast performance, especially when as-built samples contained 3 at.% Cu. Cell proliferation was only observed on ground samples without Cu but showed the lowest cell mineralization.

## 1. Introduction

The potential of titanium–copper alloys has been of high interest during the last few decades due to its multifunctionality within the biomedical field. Ti alloys, especially Ti6Al4V alloy, stand out due to their biocompatibility, mechanical properties, and excellent corrosion resistance. The addition of copper as an antibacterial element reduces the chance of bacterial infection preventing implant failure. The cytotoxicity of copper has been proven to be beneficial for killing bacteria, and it should not be harmful for the human organism if it is introduced in small doses [1]. Copper is a vital trace element to living organisms and is essential for the proper functioning of organs and metabolic processes, along with other mineral micronutrients such as iron, calcium, and zinc. Thus, copper has been studied as a promising candidate to design implant surfaces, not just for its antibacterial effects but also for its regenerative properties, since it is hypothesized that copper is able to stimulate both proliferation and osteogenic differentiation of mesenchymal stem cells [2].

For biomedical applications, Ti–Cu and Ti6Al4V–Cu alloys have been manufactured by conventional methods, such as powder metallurgy [3,4,5], arc-melting furnace [6,7,8,9,10,11,12,13,14], and by mixing elemental Ti, pre-alloyed Ti6AL4V, and Ti-Al-V powder/ingot materials with different Cu contents in the ranges 0.5–25 and 1–7.5 wt.% Cu, respectively. The percentage of copper in those alloys is important because it affects the mechanical, chemical, and biological properties of the final alloyed material. The increase in Cu content leads to an increase in antibacterial properties [7]. Bactericidal effects have been reported with the addition of 1 at.% Cu [15]. However, some authors have reported that a minimum of 5 wt.% Cu should be reached to have a strong and stable antibacterial rate [5]. The distribution and state of Cu within the microstructure provides different bactericidal results [13,14]. So far, two mechanisms have been suggested as bacteria killing mechanisms: (i) the release of Cu ions (Cu^2+^), as well as (ii) the direct Cu contact with bacteria [12,16]. Studies have shown that the minimum inhibitory Cu ion concentration against *Staphylococcus aureus* (*S. aureus*) is 448 µm/mL, while for *Escherichia coli* (*E. coli*) it is 256 µm/mL [17]. On the other hand, qualitative findings suggested that the direct interaction of Ti–Cu alloy with the bacterial cell membrane may result in enhanced permeability of the membrane, allowing entry of Cu ions into the cell, causing shrinkage of the cell membrane, and leading to cellular lysis [12]. A combination of both has been suggested in [16]. It is described that bacteria mainly exists in two forms: planctobacteria and bacteria adhered to the implant. The planctobacteria mainly react with Cu^2+^ and are killed by a charge adsorption mechanism; meanwhile, adhered bacteria that mainly react with the surface of the implant are killed by a contact mechanism [16].

However, an understanding of the effect of Cu is not limited to its study on antibacterial properties; its cell behavior also needs to be investigated to ensure proper bone osseointegration. Several studies reported that the minimum bactericidal concentration of Cu^2+^ is far lower than the minimum inhibition concentration of Cu^2+^ against cells, suggesting that copper can provide good bactericidal properties without compromising cell functioning [18]. In vitro studies show that Ti6Al4V–Cu with low Cu contents do not present cytotoxicity up to 7.5 wt.% Cu, recommending that Cu contents between 5–6% wt.% promotes good osteoblast proliferation and differentiation [11]. In vivo results also demonstrated good biocompatibility and antibacterial properties of pure Ti–10 wt.% Cu after 7 days post implantation; only mild inflammation was observed after 4 days [16].

Additive manufacturing technologies, such as laser powder bed fusion (L-PBF), provide freedom in design and offer the advantage to manufacture custom implants for the specific necessities of each customer. There have been some studies of Ti6Al4V–Cu produced by L-PBF [15,19,20,21,22]. The use of pre-alloyed Ti6Al4V powder containing either 5 wt.% elemental Cu or 0.5 wt.% elemental Ag by L-PBF was studied [19], showing that the addition of Cu had superior antibacterial properties over the addition of Ag. Krakhmalev et al. [15] reported the bactericidal properties of L-PBF Ti6Al4V(ELI)-1 at.% Cu. Results showed a notable reduction in the growth of the pure culture of *E. coli* and *S. aureus*, but the best performance was observed for higher amounts of Cu (4 and 6 wt.% Cu), which exhibited strong antibacterial properties (>90% and >99% antibacterial rate, respectively), as well as good cell cytocompatibility and better corrosion resistance in comparison with Ti6Al4V(ELI) alloy [20,21]. The addition of 6 wt.% Cu promoted osteoblast proliferation and differentiation [22]. Additionally, when manufacturing by L-PBF, the inherent surface topography of as-printed parts should be considered since it can be beneficial by means of cell response. Surface roughness plays an important role in cell attachment, as well as in cell functioning (e.g., cell proliferation, differentiation, and mineralization), which could be beneficial for osseointegration [23]. Surface roughness affects cell response at the nano- and micro-scale, but its optimal range has not been defined yet. By L-PBF, different surface roughness can be obtained depending on process parameters [24], but also depending on the orientation of the part surfaces related to the building direction, and laser scanning direction and strategy [25].

The novelty of this study focuses on the individual and combined influence of surface roughness and composition of in situ alloyed L-PBF Ti6Al4V(ELI)-3 at.% Cu material on cell response. It is of importance not only to evaluate surface features individually but also to evaluate them combined, so as to understand which parameters or combination of parameters have the most significant influence on cell behavior. Because of this, L-PBF Ti6Al4V(ELI) samples were also manufactured as a control material. The manufacturing of both materials, with and without Cu, was performed by the printing thin, vertical walls with high surface roughness. Half of the samples of each material were used in an as-built condition. The other samples were ground to achieve a smoother surface. It is important to considerer the inherent roughness of L-PBF parts, since not all components obtained by this technology (e.g., parts containing porosity or lattice structure) cannot be subjected to post-processing treatments to remove surface roughness. The present study will allow the understanding of the individual and combined effects of surface roughness and composition by means of cell morphology, viability, proliferation and cell mineralization.

## 2. Materials and Methods

### 2.1. Sample Preparation and Surface Characterization

Ti6Al4V(ELI) and in situ alloyed Ti6Al4V(ELI)-3 at.% Cu materials were produced by L-PBF, using an EOSINT M280 system (EOS GmbH, Krailling, Germany). The feedstock powders used in the present research, as well as the manufacturing process parameters and scanning strategy are described elsewhere [26,27]. For the in vitro study, thin, vertical walls of 1 mm thickness with high surface roughness were printed. Afterwards, the parts were cut into 5 × 5 × 1 mm square samples by electrical discharge machining. Side surfaces were the ones used for in-vitro assessment. After the cutting, a half of the specimens of both materials were ground with #1200 SiC paper. Two different surface roughness conditions, as-built (side surface created by layers) and ground surfaces, were studied for each material, with and without Cu, and were named as: (i) as-built Ti6Al4V(ELI), (ii) ground Ti6Al4V(ELI), (iii) as-built Ti6Al4V(ELI)-3 at.% Cu and (iv) ground Ti6Al4V(ELI)-3 at.% Cu.

Surface characterization of the samples was performed by means of surface roughness and composition. Top surfaces were observed by scanning electron microscopy (SEM, LEO 1350 FEG, Jena, Germany), operated at 20 kV, and equipped with an Oxford EDX INCA-sight system (Abingdon, UK) for chemical microanalysis. Surface roughness measurements were carried out by optical profiler Contour GT-K (Bruker, Billerica, MA, USA), in which Sa, Sz, and surface index roughness surface parameters were extracted. Sa is defined as the difference in height between the surface of each point compared to the arithmetical mean (Ra) of the surface (in absolute value). Sz is defined as the sum of the largest surface peak height value and the largest pit depth value within the defined area. Surface index is defined as an index measurement of the surface free area, “1” being a flat surface. X-ray diffraction (XRD) measurements were conducted using a Cu-Kα radiation source (wavelength λ = 0.15405 nm) operated at 40 kV and 40 mA to identify constituent phases.

### 2.2. Osteoblast Culture

Human primary osteoblasts (hOB) were obtained from the knee trabecular bone after prosthesis replacement following the protocol described by Nacher et al. [28]. The study was approved by the Parc de Salut Mar Ethics Committee. Briefly, the trabecular bone was dissected into small pieces, washed in phosphate-buffered solution (PBS), and placed into a 15 cm diameter Petri dish containing 15 mL of Dulbecco’s modified Eagle’s medium (DMEM) supplemented with 10% fetal bovine serum (FBS), penicillin (100 UI/mL), streptomycin (100 UI/mL), ascorbic acid (100 mg/mL) (Invitrogen, Waltham, MA, USA) and fungizone (0.4%) (Gibco, Waltham, MA, USA). Explants were incubated at 37 °C in a humidified atmosphere of 5% CO_2_, changing the medium once a week until cell confluence was achieved. Finally, cells were moved into new 75 cm^2^ flasks until a suitable number was reached. A maximum of a third of the subculture was used in the experiments. For materials testing, samples were sterilized overnight in ethanol at 70 °C, washed in PBS, and placed on a 48-well polystyrene culture plate (Nunc A/S). Each material was seeded with 50,000 cells and cultured with DMEM supplemented with 10% FBS and ascorbic acid; for mineralization assays, β-glycerophosphate (5 mM) was also added. Samples were tested at 1, 7, and 14 days of cell culture. For cell mineralization, samples were tested at 28 days of cell culture. Tests were carried out three times to ensure reproducibility.

### 2.3. Osteoblast Viability and Proliferation

Cell viability and proliferation were tested by MTS assay, using the CellTiter 96^®^ AQueous One Solution Cell Proliferation assay (Promega, Alcobendas, Spain), according to the manufacturer’s protocol. Fifty μL of MTS were added in each sample cultured with 250 μL of supplemented medium and incubated for 3 h. Later, the absorbance was measured at 490 nm with a scanning multi-well spectrophotometer.

The LIVE/DEAD Viability/Cytotoxicity Assay Kit for Mammalian Cells (Invitrogen, Carlsbad, CA, USA) was performed to characterize cell viability, attachment, and distribution. The test discriminates live cells from dead cells by simultaneously staining with green fluorescent calcein-AM (live cells) and red fluorescent ethidium homodimer-1 (dead cells). The LIVE/DEAD assay was performed by adding 300 μL of a solution at 4 μM EthD-1 and 2 μM of calcein AM in PBS per sample. Samples were incubated for 30–45 min at room temperature. Then the cells were observed with an Olympus BX61 microscope. Micrographs were taken and processed with Fiji software.

### 2.4. Osteoblast Mineralization Assessment

Samples were washed with PBS and fixed with 10% formalin for 10 min. Then, samples were washed with PBS and stained with 300 μL of 40 mM Alizarin red solution, with a pH of 4.2 (Sigma-Aldrich, St. Louis, MI, USA), per well at room temperature for 10 min under gentle shaking. The unincorporated dye was removed, and samples were washed carefully with PBS to remove excess stain. Then, cell mineralization was quantified by dissolving the precipitated Alizarin red with a 10% cetylpyridinium chloride solution at room temperature during 30 min on gentle shaking. One hundred μL of the stained solutions were quantified by measuring a light absorbance at 550 nm with a scanning multi-well spectrophotometer Infinite M200 (Tecan, Männedorf, Switzerland).

### 2.5. Osteoblast Morphology

Phalloidin-Tetramethylrhodamine B isothiocyanate (Sigma-Aldrich, St. Louis, MI, USA) was used for staining the structure of the cell cytoskeleton. The cells that seeded onto materials were washed with PBS twice and fixed for 10 min in a 3.7% formaldehyde (Probus, Cornwall, UK) solution in PBS. Next, cells were washed extensively in PBS and permeabilized with 0.1% TRITON^®^ X-100 (Sigma-Aldrich, St. Louis, MI, USA) in PBS for 5 min and gently rinsed with PBS. After that, cells were stained with 50 mg/mL of fluorescent phalloidin and 4′,6-diamidino phenylindole (DAPI) (0.2 mg/mL) (Sigma-Aldrich, St. Louis, MI, USA) in PBS (protected from the light) for 40 min at room temperature. Cells were observed with the Olympus BX61 microscope, and the micrographs were processed with Fiji software.

### 2.6. Statistical Analysis

Statistical analyses were performed by the Wilcoxon–Mann–Whitney test in the SPSS v.12.0 for Windows. Differences with a * *p*-value < 0.05 were considered statistically significant. All tests were carried out three times with independent cell lines to ensure reproducibility. Each test contained three replicas of each sample and was tested together with positive and negative controls. The results were normalized by the ground L-PBF Ti6Al4V(ELI) condition within each experiment and each time to reduce the inter-experiment variability. The normalization of the results allowed a values comparison between different days and repeatability of experiments where different cell lines were used in each experiment.

## 3. Results

### 3.1. Surface Topography

**Figure 1:**
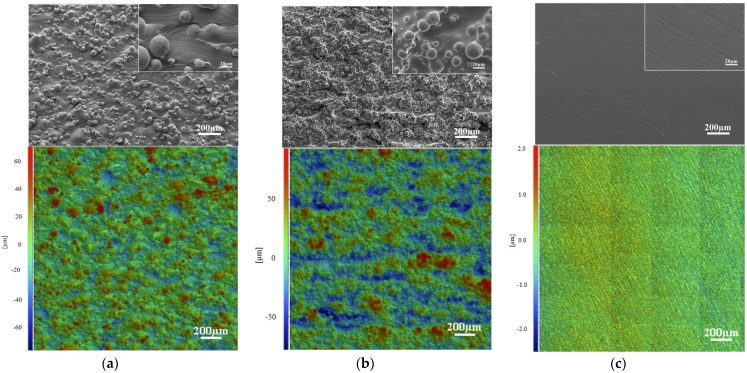
SEM and surface topography micrographs of (**a**) as-built L-PBF Ti6Al4V(ELI), (**b**) Ti6Al4V(ELI)-3 at.% Cu surfaces, and (**c**) after grinding process (same surface topography was obtained for both samples with and without 3 at.% Cu).

Figure 1 shows the SEM micrographs and surface topography measurements of ground and as-built Ti6Al4V(ELI) and Ti6Al4V(ELI)-3 at.% Cu side surfaces. As-built surfaces showed a higher surface roughness in comparison with the ground ones due to the partially melted particles of the L-PBF process (Figure 1a,b). A higher amount of partially melted and un-melted attached particles was observed on Ti6Al4V(ELI)-3 at.% Cu surfaces in comparison with Ti6Al4V(ELI). Surface roughness measurements showed the same evidence with a higher distance between surface peaks and valleys. Table 1 shows the surface roughness measurements of the studied specimens. No significant differences were found between both as-built surfaces, with and without 3 at.% Cu (Sa = 17.6 ± 0.6 µm and 12.3 ± 0.7 µm, respectively). As-built surfaces had a higher surface index due to the higher surface roughness in comparison with ground surfaces. After the grinding process of as-built samples with and without 3 at.% Cu, surface roughness reduced from Sa ≈ 12–17 µm (as-built surface) to Sa ≈ 0.5 µm (ground surface), as well as the surface index from ~1.82 to ~1.02. Figure 1c shows that the ground surface has a one direction parallel scratch pattern, due to the grinding process. The same surface characteristic was present on ground samples with and without 3 at.% Cu.

### 3.2. Microstructure and Surface Composition

**Figure 2:**
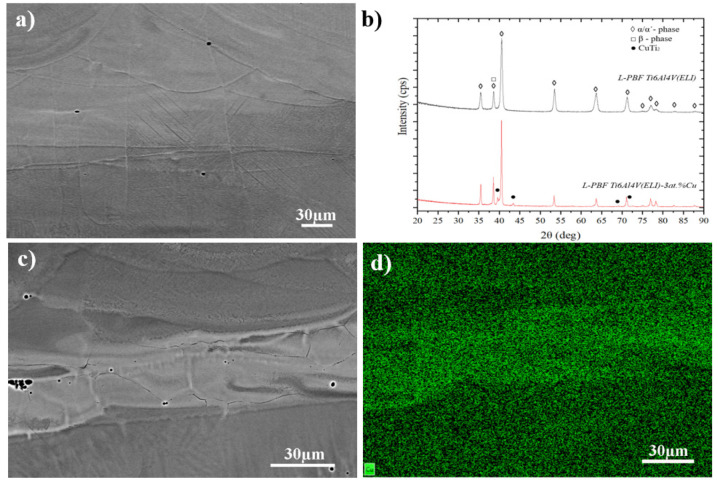
SEM BSE micrographs of the central area of L-PBF Ti6Al4V(ELI)-3 at.% Cu specimen, (**a**) illustrating general microstructure, (**b**) XRD (with L-PBF Ti6Al4V(ELI) as reference), (**c**) Cu-rich region and (**d**) Cu map of (**c**).

Figure 2a,b show the chemical homogeneity of L-PBF Ti6Al4V(ELI)-3 at.% Cu material. SEM micrographs were taken in back-scattered electron (BSE) mode. The in situ alloyed material displayed a chemical inhomogeneity where Cu tended to segregate at the fusion border of the three-dimensional printed tracks. Generally, the average of Cu in the material was 2.1–3.5 wt.%; meanwhile the fusion boundaries reached values between 19–23 wt.% [27]. Both Ti6Al4V(ELI) and Ti6Al4V(ELI)-3 at.% Cu materials showed a fine-needle α’ martensite microstructure which has been previously reported in the literature. XRD results showed that L-PBF Ti6Al4V(ELI) material was composed by α’martensite phase and possible β phase. It is hard to determine β phase by XRD due to the peak overlapping of cubic β phase <110> with α/α’-phase <002> (Figure 2c). However, the addition of Cu as a β stabilizer can increase the percentage of β phase. This can be observed in the slight increase in intensity of the overlapped α/α’- β phase peak (Figure 2b). Additionally, the addition of Cu led to the precipitation of the CuTi_2_ intermetallic phase (Figure 2c). Because of the differences in chemical composition in different areas, galvanic pairs may form, resulting in selective corrosion of some regions. However, further work is required to determine if selective corrosion occurs and if it is critical or not.

### 3.3. Osteoblast Viability and Proliferation

**Figure 3:**
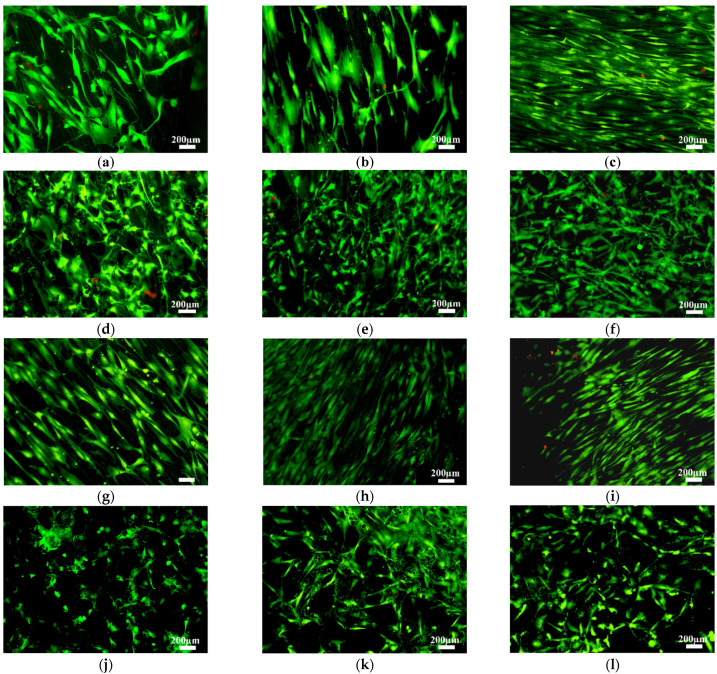
Fluorescence images of a LIVE/DEAD assay of osteoblast cells cultured for 1, 7, and 14 days (from left to right) of cell culture onto (**a**–**c**) ground and (**d**–**f**) as-built L-PBF Ti6Al4V(ELI) and, (**g**–**i**) ground and (**j**–**l**) as-built L-PBF Ti6Al4V(ELI)-3 at.% Cu surfaces (*n* = 3).

**Figure 4:**
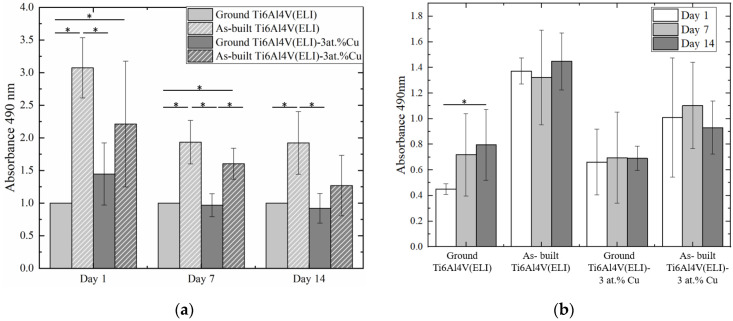
MTS assay at 1, 7, and 14 days of cell culture onto ground and as-built L-PBF Ti6Al4V(ELI) and Ti6Al4V(ELI)-3 at.% Cu surfaces. (**a**) Normalized MTS values in each tested time and, (**b**) non-normalized MTS results per studied materials (*n* = 3; * *p*-values < 0.05).

LIVE/DEAD assays were used to test the cytotoxicity of materials at 1, 7, and 14 days of cell culture. None of the materials showed a significant number of dead cells (red color, Figure 3), indicating a good cytocompatibility of the in situ alloyed material. MTS cell viability results are presented with and without normalization at 1, 7, and 14 days of cell culture. The normalized results in Figure 4a allows a better comparison of the number of viable cells among materials at each studied day; meanwhile, non-normalized values in Figure 4b allow the analysis of cell proliferation by comparing the number of cells on each material among the different tested times. At 1 day of cell culture, a higher number of cells were deposited onto as-built surfaces (rougher) than on the ground (smoother) surfaces for both materials, with and without 3 at.% Cu content (Figure 4a). The difference between ground and as-built Ti6Al4V(ELI) surfaces was significant, showing cell deposition three times higher on as-built surfaces and suggesting a higher initial cell attachment due to the increase in surface roughness. However, the increase in initial cell deposition due to surface roughness was not significant when Cu was added, although the average value of deposited cells on as-built Ti6Al4V(ELI)-3 at.% Cu surfaces was higher than ground Ti6Al4V(ELI)-3 at.% Cu surface ones. At 7 days of cell culture, a significant number of cells was observed on as-built surfaces, both with and without Cu content (Figure 4a). At 14 days of cell culture, as-built Ti6Al4V(ELI) had the largest number of deposited cells. No significant differences in cell number were observed due to the addition of 3 at.% Cu, or with the combination of it together with the increase in surface roughness. Results showed that cell deposition was most strongly influenced by surface roughness rather than composition in the present study (Figure 4a).

Figure 4b shows the results of cell proliferation for each material from 1 to 14 days of cell culture. No cell proliferation was observed on as-built nor 3 at.% Cu samples. The ground Ti6Al4V(ELI) surface was the only one that showed cell proliferation at 14 days of cell culture. It was corroborated by LIVE/DEAD micrographs in which only ground Ti6Al4V(ELI) showed a cell number increase between 1 and 14 days of cell culture (Figure 3a–c).

### 3.4. Osteoblast Morphology

**Figure 5:**
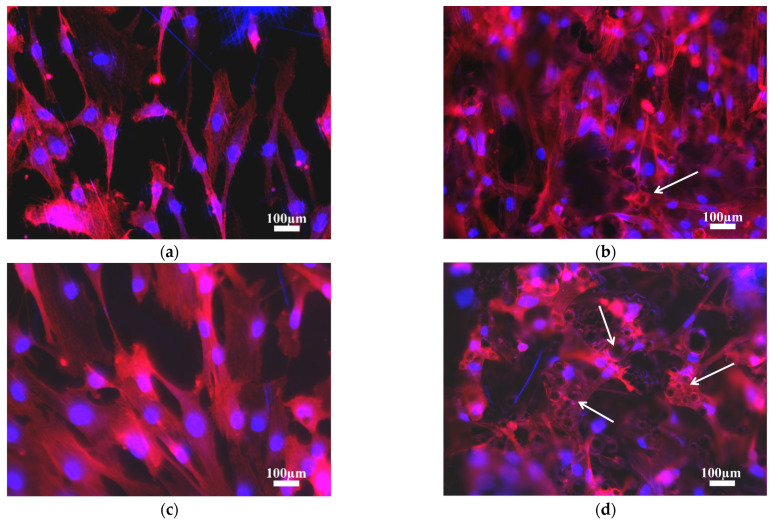
Phalloidin staining at 1 day of cell culture onto (**a**) ground and (**b**) as-built L-PBF Ti6Al4V(ELI) and (**c**) ground and (**d**) as-built L-PBF Ti6Al4V(ELI)-3 at.% Cu surfaces (*n* = 3). White arrows show the cell cytoplasm covering several surface peaks formed due to the attachment of un-melted or partially melted particles of as-built surfaces.

**Figure 6:**
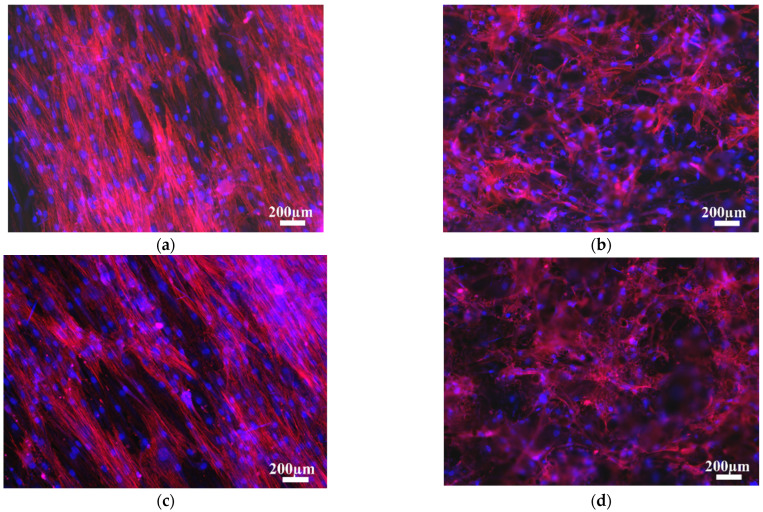
Phalloidin staining at 14 days of cell culture onto (**a**) ground and (**b**) as-built L-PBF Ti6Al4V(ELI) and (**c**) ground and (**d**) as-built L-PBF Ti6Al4V(ELI)-3 at.% Cu surfaces (*n* = 3).

Cell morphology at 1 day of cell culture was studied by phalloidin cell staining (Figure 5). Different cell morphologies were distinguished based on surface topography, independently of 3 at.% Cu content. Cells on ground surfaces showed flattened and elongated cytoplasm due to the lower surface roughness, and were influenced by surface morphology, orientating themselves along the scratch parallel pattern from the grinding process (Figure 5a,c). Cells on as-built surfaces showed more polygonal extended filopodia due to the higher surface roughness, but also had random orientation of peaks and valleys. Figure 5d show cell cytoplasm covering several surface peaks formed due to the attachment of un-melted or partially melted particles on the as-built L-PBF surface (Figure 5, white arrows). At 14 days of cell culture, cells showed the same cell morphology and orientation as at 1 day (Figure 6). Cells on ground surfaces showed elongated cytoplasm, as well as good connectivity between cells (Figure 6a,c). On the other hand, as-built surfaces kept showing extended cytoplasm due to higher surface roughness, and went in several directions due the random surface morphology (Figure 6b,d).

### 3.5. Osteoblast Function

**Figure 7:**
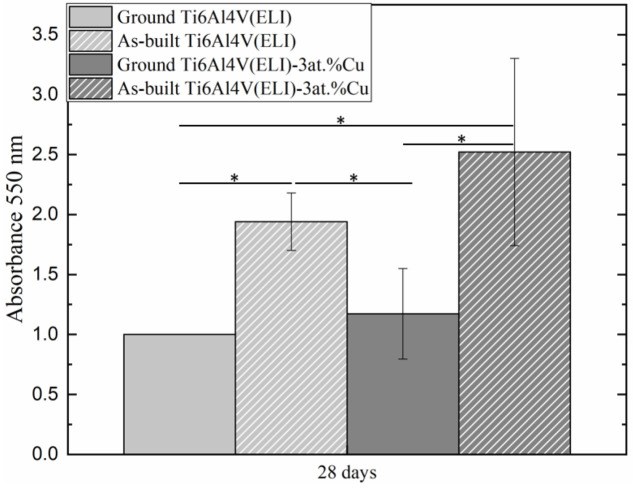
Cell mineralization assay at 28 days of cell culture onto ground and as-built L-PBF Ti6Al4V(ELI) and Ti6Al4V(ELI)-3 at.% Cu (*n* = 3; * *p*-values < 0.05).

Cell mineralization capacity was evaluated at 28 days of cell culture. It was observed that as-built surfaces had significantly higher mineralization than the ground ones, regardless of Cu addition (Figure 7). The addition of 3 at.% Cu did not significantly influence osteoblast performance. However, the increase in cell mineralization was observed in the material with 3 at.% Cu content, especially on higher surface roughness (as-built Ti6Al4V(ELI)-3 at.% Cu surfaces).

## 4. Discussion

The present study focuses on the individual and combined effects on cell behavior of surface roughness and surface composition of in situ alloyed Ti6Al4V(ELI)-3 at.% Cu material obtained by the L-PBF technique. The discussion is based on cell behavior by means of cell attachment, viability, proliferation, and mineralization capacity.

### 4.1. Osteoblast Attachment

The combination of Ti alloys with Cu, such as Ti–Cu and Ti6Al4V–Cu, can result in a good combination of mechanical and biological properties that are promising for implant applications due to the increase in strength and hardness of the material and by providing good cytocompatibility with the organism [27,29]. After implantation, a rapid interaction takes place between the implant and the surrounding tissue and therefore, the initial cell attachment takes particular relevance. For ground Ti–Cu alloy samples obtained by powder metallurgy, MG63 cells showed good cell adhesion and spreading on the material for Cu contents up to 25 wt.% [30]. No differences have been reported in cell morphology between pure-Ti and Ti–Cu materials, indicating that Cu samples did not interfere in cell morphology after 1 day of cell culture. The same findings were found with ground Ti- 5 wt.% Cu samples produced by vacuum arc melting, and MG63 cells extended filopodia following the surface scratches from the ground surface [31].

In the current research, Ti6Al4V(ELI) and Cu powders were in situ alloyed and produced by the L-PBF technique. Cells on the ground L-PBF Ti6Al4V(ELI)- 3 at.% Cu material showed good cell attachment with cell spreading and filopodial extensions. No differences in cell attachment were found between ground L-PBF Ti6Al4V(ELI) surfaces with or without 3 at.% Cu content. The same findings were reported by Luo et al. [22] and Xu et al. [20] between L-PBF Ti6AlV and Ti6Al4V-6 wt.% Cu for MG 63 and HUVECx cells, respectively, and also between Ti6Al4V and Ti6Al4V-*x*Cu (*x* = 1, 3, 5 wt.% Cu) material obtained by vacuum arc melting [7]. However, in this study, the initial cell deposition was observed to be significantly higher on as-built surfaces versus ground surfaces, with higher surface roughness independently from 3 at.% Cu content. No differences were found between both as-built surfaces. This fact indicated that 3 at.% Cu content did not negatively influence the initial osteoblast cell attachment, and that the surface roughness was the most significant parameter. Although not significant, the addition of 3 at.% Cu in as-built Ti6Al4V(ELI) resulted in a lower cell deposition average value than was observed for the same material without Cu. At the same time, both values were higher than those observed for ground Ti6Al4V(ELI)-3 at.% Cu. The surface index revealed that the increase in free surface area of as-built L-PBF specimens was almost double than that of the ground L-PBF specimens. The increase in free surface area led to larger beneficial areas for the increase of cell deposition, but at the same time, led to larger areas containing higher concentrations of Cu, which potentially increased Cu^2+^ release. Burghardt et al. [2] has reported a concentration of 0.5 mM as a threshold for toxic effects of copper ions for mesenchymal stem cells (MSC), in which cells became rounded and detached from the surface when they were exposed to this concentration for 14 days of cell culture. Thus, an increase in Cu^2+^ content may negatively affect cell deposition locally. However, further studies of metal ion release should be performed, as well as for Ti-Cu and Ti6Al4V-Cu alloys, since the release of Al and V ions is known to promote moderate and high cell cytotoxicity, respectively [23]. It has been reported that the increase in Cu content in Ti6AL4V-*x*Cu alloys, resulted not only in an increase in Cu ions, but also increased a release in Ti and Al ions too [22].

### 4.2. Osteoblast Proliferation and Mineralization

Literature contains contradictory information about the effect of Cu-containing surfaces on osteoblast. There is uncertainty about the effect Cu has on cell behavior. In the present study, cell proliferation was only observed on ground Ti6Al4V(ELI) surfaces after 14 days of cell culture. Results showed that cell proliferation was inhibited when surface roughness was increased (as-built surfaces) or 3 at.% Cu content was added. Similar research has been observed on ground L-PBF Ti6Al4V-6 wt.% Cu that showed an increase in MG63 and rat osteoblasts proliferation up to 3 and 7 days of cell culture, respectively, but no differences were found between samples with and without copper for each studied day [20,22]. These results agreed with Zhang et al. [30], in which no difference in cell proliferation was found between pure Ti and Ti-25 wt.% Cu for up to 3 days. Further studies should be performed to clarify the effect of microstructure on cell behavior, since different manufacturing processes led to different phases and microstructures. Depending on what phase the Cu ions are released, for example, from a solid solution or a chemical compound, different release rates can be observed. Under equilibrium conditions, α and β phases coexist together with CuTi_2_ intermetallic in Ti6Al4V-Cu alloys [27]. Different manufacturing processes, as well as different process parameters, significantly affect the microstructure, leading to different percentages of phases, or differences in the CuTi_2_ intermetallic precipitates size. Ma et al. [14] studied the effects of heat treatment of Ti6Al4V-5 wt.% Cu, which they obtained using an arc melting furnace, on Cu distribution and its bactericidal and cellular response, reporting that Cu can exist in two different states: interstitial solid solution in the α and β phase and precipitation of intermetallic CuTi_2_ compound. The release of Cu ions is easier from the alloy for interstitial solid solution than from stable CuTi_2_ phase. Thus, observations have shown that higher solution treatment temperatures exhibited better antibacterial properties due to a higher amount of β phase formed in the microstructure, since Cu is prone to mainly exist in β phase as a β stabilizer in titanium [14]. Burghardt et al. [2] has reported that the increase in Cu^2+^ ions has stimulated MSC proliferation up to an optimal concentration of 0.1 mM Cu^2+^. However, cell proliferation is reduced at higher Cu^2+^ release (0.3 mM).

Cell mineralization levels were higher for as-built surfaces that, along with the lack of cell proliferation, were compatible with a higher cell differentiation status. This increase in the production of extracellular matrices with the increase in surface roughness is corroborated by the literature [23]. Although the detailed mechanism still requires further study, it has been demonstrated that larger cell spreading is beneficial for osteoblast differentiation [22]. On the other hand, the addition of 3 at.% Cu content in ground surfaces has not provided significant changes in the production of the extracellular matrix. Luo et al. [22] reported a gradual increase in MG63 cell differentiation values with the increase in Cu content in ground L-PBF Ti6Al4V-Cu (*x* = 2, 4, 6 wt.% Cu) material. However, the differences were not reported as significant when the tested samples contained 2 or 4 wt.% Cu, but the difference has been significant when the alloy contained 6 wt.% Cu compared to Ti6Al4V. On the other hand, Xu et al. [20] reported no differences in cell mineralization between ground L-PBF Ti6Al4V-6 wt.% and pure Ti6Al4V alloy. The same cell mineralization behavior was found in the present study in the L-PBF Ti6Al4V(ELI) with 3 at.% Cu (equivalent to 4.1 wt.% Cu). However, in the present study, the combination of surface roughness together with 3 at.% Cu conferred the highest cell mineralization average values at 28 days of cell culture. A possible explanation could be the increase in free surface area due to the increase in the surface roughness, which is linked to the increase in the release of Cu^2+^, thus enhancing osteogenic activity. However, deeper analysis of Cu distribution among phases in the alloy, as well as Cu^2+^ release rates from different phases, should be performed.

## 5. Conclusions

In the present research, the influence of surface roughness and chemical composition on cell response of in situ alloyed L-PBF Ti6Al4V(ELI)-3 at.% Cu was investigated, both individually and combined. A surface topography comparison between ground and as-built L-PBF surfaces, with and without 3 at.% Cu, was performed.

Good cell attachment was observed and no cytotoxic effects were found for any of the studied conditions for the entire duration days of cell culture, indicating the non-cytotoxicity of the L-PBF Ti6Al4V(ELI) with 3 at.% Cu content. As-built Ti6Al4V(ELI) surfaces had higher cell deposition than ground ones due to their larger surface area. The as-built L-PBF surface roughness inhibited cell proliferation but significantly increased cell mineralization capacity, especially in combination with 3 at.% Cu content. Ground Ti6Al4V(ELI) surfaces were the only ones that showed cell proliferation at 14 days of cell culture, since the addition of 3 at.% Cu content was observed to inhibit cell proliferation. Further studies on the release of metal ions should be performed to better understand the effect of copper on cell behavior, especially in combination with other parameters such as surface roughness.

The presented results confirm the promising combination of surface roughness with the addition of 3 at.% Cu for higher cell performance of Ti64-Cu materials for implants. Nevertheless, for a better understanding and to avoid unexpected harmful effects, more investigations regarding differences in chemical composition, formation of galvanic pairs, and distribution of Cu within Ti6Al4V matrix are needed. The results of this new material performance are really promising, but at the same time, they are not directly applicable in hospitals. Therefore, more investigations are suggested.

## Figures and Tables

**Table 1:** Surface roughness parameters of the L-PBF studied specimens.

	Surface Roughness Parameters		
	Sa (µm)	Sz (µm)	Surface Index (-)
Ground Ti6Al4V(ELI)/Ti6Al4V(ELI)-3 at.% Cu	0.5 ± 0.1	15.8 ± 6.9	1.02 ± 0.1
As-built Ti6Al4V(ELI)	12.3 ± 0.7	177.5 ± 25.2	1.83 ± 0.1
As-built Ti6Al4V(ELI)-3 at.% Cu	17.6 ± 0.6	193.5 ± 16.2	1.81 ± 0.1

## Data Availability

The data presented in this study are available on request from the corresponding author.

## References

[1] Copper: Essential for Human Health. https://copperalliance.org.uk/knowledge-base/education/education-resources/copper-essential-human-health/.

[2] Burghardt I., Lüthen F., Prinz C., Kreikemeyer B., Zietz C., Neumann H.-G., Rychly J. (2015). A dual function of copper in designing regenerative implants. Biomaterials.

[3] Alshammari Y., Yang F., Bolzoni L. (2019). Low-cost powder metallurgy Ti-Cu alloys as a potential antibacterial material. J. Mech. Behav. Biomed..

[4] Zhang E., Li F., Wang H., Liu J., Wang C., Li M., Yang K. (2013). A new antibacterial titanium–copper sintered alloy: Preparation and antibacterial property. Mater. Sci. Eng. C.

[5] Liu J., Li F., Liu C., Wang H., Ren B., Yang K., Zhang E. (2014). Effect of Cu content on the antibacterial activity of titanium–copper sintered alloys. Mater. Sci. Eng. C.

[6] Fowler L., Janson O., Engqvist H., Norgren S., Öhman-Mägi C. (2019). Antibacterial investigation of titanium-copper alloys using luminescent Staphylococcus epidermidis in a direct contact test. Mater. Sci. Eng. C.

[7] Ren L., Ma Z., Li M., Zhang Y., Liu W., Liao Z., Yang K. (2014). Antibacterial Properties of Ti–6Al–4V–*x*Cu Alloys. J. Mater. Sci. Technol..

[8] Aoki TOkafor I.C.I., Watanabe I., Hattori M., Oda Y., Okabe T. (2004). Mechanical properties of cast Ti-6Al-4V-XCu alloys. J. Oral. Rehabil..

[9] Souza S.A., Afonso C.R.M., Ferrandini P.L., Coelho A.A., Caram R. (2009). Effect of cooling rate on Ti–Cu eutectoid alloy microstructure. Mater. Sci. Eng. C.

[10] Kikuchi M., Takada Y., Kiyosue S., Yoda M., Woldu M., Cai Z., Okuno O., Okabe T. (2003). Mechanical properties and microstructures of cast Ti–Cu alloys. Dental. Mater..

[11] Peng C., Liu Y., Liu H., Zhang S., Bai C., Wan Y., Ren L., Yang K. (2019). Optimization of annealing treatment and comprehensive properties of Cu-containing Ti6Al4V-*x*Cu alloys. J. Mater. Sci. Technol..

[12] Liu R., Memarzadeh K., Chang B., Zhang Y., Ma Z., Allaker R.P., Ren L., Yang K. (2016). Antibacterial effect of copper-bearing titanium alloy (Ti-Cu) against Streptococcus mutans and Porphyromonas gingivalis. Sci. Rep..

[13] Zhang E., Wang X., Chen M., Hou B. (2016). Effect of the existing form of Cu element on the mechanical properties, bio-corrosion and antibacterial properties of Ti-Cu alloys for biomedical application. Mater. Sci. Eng. C.

[14] Ma Z., Ren L., Liu R., Yang K., Zhang Y., Liao Z., Liu W., Qi M., Misra R.D.K. (2015). Effect of Heat Treatment on Cu Distribution, Antibacterial Performance and Cytotoxicity of Ti–6Al–4V–5Cu Alloy. J. Mater. Sci. Technol..

[15] Krakhmalev P., Yadroitsev I., Yadroitsava I., de Smidt O. (2017). Functionalization of Biomedical Ti6Al4V via In Situ Alloying by Cu during Laser Powder Bed Fusion Manufacturing. Materials.

[16] Wang X., Dong H., Liu J., Qin G., Chen D., Zhang E. (2019). In vivo antibacterial property of Ti-Cu sintered alloy implant. Mater. Sci. Eng. C.

[17] Du W.L., Niu S.S., Xu Y.L., Xu Z.R., Fan C.L. (2009). Antibacterial activity of chitosan tripolyphosphate nanoparticles loaded with various metal ions. Carbohydr. Polymers..

[18] Zhang E.L., Fu S., Wang R.X., Li H.X., Liu Y., Ma Z.Q., Liu G.K., Zhu C.S., Qin G.W., Chen D.F. (2019). Role of Cu element in biomedical metal alloy design. Rare Met..

[19] Macpherson A., Li X., McCormick P., Ren L., Yang K., Sercombe T.B. (2017). Antibacterial Titanium Produced Using Selective Laser Melting. JOM.

[20] Xu X., Lu Y., Li S., Guo S., He M., Luo K., Lin J. (2018). Copper-modified Ti6Al4V alloy fabricated by selective laser melting with pro-angiogenic and anti-inflammatory properties for potential guided bone regeneration applications. Mater. Sci. Eng. C.

[21] Guo S., Lu Y., Wu S., Liu L., He M., Zhao C., Gan Y., Lin J., Luo J., Xu X. (2017). Preliminary study on the corrosion resistance, antibacterial activity and cytotoxicity of selective-laser-melted Ti6Al4V- x Cu alloys. Mater. Sci. Eng. C.

[22] Luo J., Guo S., Lu Y., Xu X., Zhao C., Wu S., Lin J. (2018). Cytocompatibility of Cu-bearing Ti6Al4V alloys manufactured by selective laser melting. Mater. Charact..

[23] Vilardell A.M., Cinca N., Garcia-Giralt N., Dosta S., Cano I.G., Nogués X., Guilemany J.M. (2018). Osteoblastic cell response on high-rough titanium coatings by cold spray. J. Mater. Sci. Mater. Med..

[24] Charles A., Elkaseer A., Thijs L., Hagenmeyer V., Scholz S. (2019). Effect of Process Parameters on the Generated Surface Roughness of Down-Facing Surfaces in Selective Laser Melting. Appl. Sci..

[25] Vilardell A.M., Krakhmalev P., Fredriksson G., Cabanettes F., Sova A., Valentin D., Bertrand P. (2018). Influence of surface topography on fatigue behavior of Ti6Al4V alloy by laser powder bed fusion. Procedia CIRP.

[26] Vilardell A.M., Takezawa A., du Plessis A., Takata N., Krakhmalev P., Kobashi M., Yadroitsava I., Yadroitsev I. (2019). Topology optimization and characterization of Ti6Al4V ELI cellular lattice structures by laser powder bed fusion for biomedical applications. Mater. Sci. Eng. A.

[27] Vilardell A.M., Yadroitsev I., Yadroitsava I., Albu M., Takata N., Kobashi M., Krakhmalev P., Kouprianoff D., Kothleitner G., du Plessis A. (2020). Manufacturing and characterization of in-situ alloyed Ti6Al4V(ELI)-3 at.% Cu by laser powder bed fusion. Addit. Manuf..

[28] Nàcher M., Aubia J., Serrano S., Mariñoso M.L., Hernández J., Bosch J., Díez A., Puig J.M., Lloveras J. (1994). Effect of cyclosporine A on normal human osteoblasts in vitro. Bone Miner..

[29] Vilardell A.M., Takezawa A., du Plessis A., Takata N., Krakhmalev P., Kobashi M., Albu M., Kothleitner G., Yadroitsava I., Yadroitsev I. (2021). Mechanical behavior of in-situ alloyed Ti6Al4V(ELI)-3 at.% Cu lattice structures manufactured by laser powder bed fusion and designed for implant applications. J. Mech. Behav. Biomed. Mater..

[30] Zhang E., Zheng L., Liu J., Bai B., Liu C. (2015). Influence of Cu content on the cell biocompatibility of Ti–Cu sintered alloys. Mater. Sci. Eng. C.

[31] Liu R., Ma Z., Kunle Kolawole S., Zeng L., Zhao Y., Ren L., Yang K. (2019). In vitro study on cytocompatibility and osteogenesis ability of Ti–Cu alloy. J. Mater. Sci. Mater. Med..

